# Cellular Efficacy of Fattigated Nanoparticles and Real-Time ROS Occurrence Using Microfluidic Hepatocarcinoma Chip System: Effect of Anticancer Drug Solubility and Shear Stress

**DOI:** 10.3390/ph16091330

**Published:** 2023-09-20

**Authors:** Hoyoung Kim, Eun-Ji Kim, Hai V. Ngo, Hy D. Nguyen, Chulhun Park, Kyung Hyun Choi, Jun-Bom Park, Beom-Jin Lee

**Affiliations:** 1 College of Pharmacy, Ajou University, Suwon 16499, Republic of Korea; khwoomg@naver.com (H.K.); omo5453@naver.com (E.-J.K.); haingo25895@gmail.com (H.V.N.); dinhhyd14@gmail.com (H.D.N.); 2 College of Pharmacy, Jeju National University, Jeju 63243, Republic of Korea; chulhunp1020@gmail.com; 3 Advanced Micro-Mechatronics Lab, Mechatronics Engineering, Jeju National University, Jeju 63243, Republic of Korea; amm3713@gmail.com; 4 BioSpero, Jeju 63309, Republic of Korea; 5 College of Pharmacy, Sahmyook University, Seoul 01795, Republic of Korea; junji4@gmail.com

**Keywords:** fattigated nanoparticles, biomimetic microfluidic system, cellular viability, real-time ROS sensor chip, shear stress, drug solubility

## Abstract

The objective of this study was to evaluate the effectiveness of organ-on-chip system investigating simultaneous cellular efficacy and real-time reactive oxygen species (ROS) occurrence of anticancer drug-loaded nanoparticles (NPs) using hepatocarcinoma cells (HepG2) chip system under static and hepatomimicking shear stress conditions (5 dyne/cm^2^). Then, the role of hepatomimetic shear stress exposed to HepG2 and drug solubility were compared. The highly soluble doxorubicin (DOX) and poorly soluble paclitaxel (PTX) were chosen. Fattigated NPs (AONs) were formed via self-assembly of amphiphilic albumin (HSA)-oleic acid conjugate (AOC). Then, drug-loaded AONs (DOX-AON or PTX-AON) were exposed to a serum-free HepG2 medium at 37 °C and 5% carbon dioxide for 24 h using a real-time ROS sensor chip-based microfluidic system. The cellular efficacy and simultaneous ROS occurrence of free drugs and drug-loaded AONs were compared. The cellular efficacy of drug-loaded AONs varied in a dose-dependent manner and were consistently correlated with real-time of ROS occurrence. Drug-loaded AONs increased the intracellular fluorescence intensity and decreased the cellular efficacy compared to free drugs under dynamic conditions. The half-maximal inhibitory concentration (IC_50_) values of free DOX (13.4 μg/mL) and PTX (54.44 μg/mL) under static conditions decreased to 11.79 and 38.43 μg/mL, respectively, under dynamic conditions. Furthermore, DOX- and PTX-AONs showed highly decreased IC_50_ values of 5.613 and 21.86 μg/mL, respectively, as compared to free drugs under dynamic conditions. It was evident that cellular efficacy and real-time ROS occurrence were well-correlated and highly dependent on the drug-loaded nanostructure, drug solubility and physiological shear stress.

## 1. Introduction

During drug development, preclinical testing is a valuable strategy for predicting outcomes by accurately evaluating the efficacy and toxicity of drugs via complex in vivo interactions. Animal models are used in preclinical testing to identify the pharmacodynamic and pharmacokinetic properties of drugs. Commonly used animal models include mice, rats, rabbits, dogs, pigs, and monkeys [1]. These animal models show characteristics that closely resemble human phenomena because their essential body components and main functions necessary for maintaining life are identical to those of humans. In addition, the genomic identity of the protein-coding region is 95–99% similar in humans and chimpanzees and 85% similar in humans and mice [2,3]. As genetic similarity is high, disease progression and physiological responses are also similar, making it a suitable evaluation model of pharmacokinetics and pharmacodynamics for the development of therapeutic agents. However, the biochemical and biopharmaceutical properties of animal models, such as metabolism and immune responses, differ from those of humans. For example, thalidomide toxicity was not observed in small rodents, whereas fatal side effects were observed in humans [4]. In addition, using an animal model during preclinical tests significantly increases the total cost and ethical issues of developing a new drug [5]. Therefore, there is a need to improve these platforms to develop more efficient and economical experimental models that can mimic in vivo pharmacokinetic and pharmacodynamic profiles [6,7,8].

Biomimetic and microfluidic chip models using human-oriented tissues and organs to replace animal testing are being tried in various ways. This can be achieved using animal-alternative biomimetic chip systems, such as a cellular culture model derived from the human body to evaluate the drug efficacy and toxicity in various real-time chip sensors [9]. Disease cells derived from the patient’s organs (heart, kidney, liver, etc.) are also cultured in biomimetic microfluidic chips. The disease model chip implemented can be utilized to determine the therapeutic efficacy and long-term toxicity of the drug against the disease. It can also be used to predict the biological behavior of drugs by connecting each organ chip to one system [10]. In the microenvironment, the cells are subjected to pressurize through the fluidic shear stress, which varies cellular behaviors than that in a static culture environment, thereby promoting cell membrane transport and metabolism [11]. It is known that living cells possess the ability to sense mechanical stimulus by fluid shear stress and transduce those into biological responses, that is absent in static culture systems. Hepatocytes cultured in a microfluidic environment can undergo more biomimetic conditions like those in vivo, mimicking liver tissues and enabling the evaluation of drug efficacy and toxicity [12,13].

In addition, there are many attempts to monitor the response of the microfluidic organ chip systems to the drugs in real-time in the organ mimicking chip by fusing the chip and sensing technology [14,15,16]. Among these attempts, sensing research on oxidative stress, which can check cytotoxicity and the response of cells to drugs, is being actively conducted [17,18]. This is because unstable and short-lived molecules such as ROS and reactive nitrogen species (RNS) derived from cells by drugs and external stimuli through electrochemical methods can be detected by sensors before they occur and disappear from cells. There is also an advantage in that the reliability of the sensor may be guaranteed by varying the selection of electrodes depending on the type of ROS and RNS [19]. This environment uses ROS sensors to monitor real-time cell responses by drugs, enabling rapid evaluation of in vitro cellular performances.

This study aimed to simultaneously evaluate the cellular efficacy and real-time ROS sensing data by exposing free anticancer model drugs with different solubility and drug-loaded AONs under static and hepatomimetic dynamic environments (fluidic liver shear stress, 5 dyne/cm^2^) to cultured HepG2 chip system. Highly water-soluble DOX and practically water-insoluble PTX were chosen as model drugs. The physicochemical properties of AONs and drug-loaded AONs, including particle size, zeta potential, drug loading content, encapsulation efficiency, and surface morphology using a scanning electron microscope, were investigated. Based on data of cellular viability, the half-maximal inhibitory concentration (IC_50_) values of free drug and drug-loaded AONs were compared according to the drug types and shear stress. Finally, the correlation between cellular efficacy and real-time ROS occurrence as a function of drug concentrations was validated.

## 2. Results and Discussion

### 2.1. Identification of AOC and Physicochemical Properties of AONs

#### 2.1.1. Identification of AOCs

The bonding process between HSA and OA by EDC-NHS coupling having the -COOH of OA activated by EDC and sulfo-NHS was conjugated to the free amine group of HSA. The stepwise synthetic process and identification of fattigated nanoparticles (AONs), including X-ray powder diffraction (XRD), differential scanning calorimetry (DSC), time-of-flight mass spectrometry (MALDI/TOF), circular dichroism (CD) spectra were thoroughly documented in previous publications [20]. Figure S2 shows the FT-IR spectra of OA, HSA, physical mixture (HSA and OA), and AOC. The wavenumber change of these peaks was induced along with the N-H stretch band at 3290 and 1534 cm^−1^ by a reaction between HSA and OA in the FT-IR spectra. In addition, we observed a characteristic absorption peak at 2850–2950 cm^−1^, corresponding to the CH_2_ group included in OA. This was indicated as a band moving from 1644 to 1649 cm^−1^ by the formation of an amide bond between the amino group of HSA and the NHS ester group of OA generated through EDC/NHS coupling. This showed a shift in the absorption peak corresponding to the C=O stretch of the secondary amide. The physicochemical structure of the particles was changed through a chemical reaction between the functional groups of HSA and OA, and the conjugates were successfully formed.

#### 2.1.2. Physicochemical Properties of AONs

The physicochemical properties of NPs, such as particle size and zeta potential, had a significant effect on cellular uptake. That is, the smaller the size, the higher the uptake efficiency [21]. Zeta potential also has a significant effect on cellular uptake. The cell membrane potential of the liver cancer cell line HepG2 used in this study was approximately −20 mV [22]. The surface charge of AONs is related to their binding to the cell membrane and how they are absorbed into the cell. A higher positivity of membrane potential enables easier binding and absorption processes. However, the nanoparticle stability was determined by the zeta potential. NPs maintain a stable state if the surface charge of the particles is −30 mV or more [23].

The physicochemical properties of the self-assembled AONs with or without drug loadings are listed in Table 1. Figure S3 also gives the particle distribution of AONs (181.20 ± 29.20 nm), DOX-AONs (313.23 ± 3.97 nm), and PTX- AONs (438.90 ± 27.97 nm). The particle size increased in the order of AONs, DOX-AONs, and PTX-AONs. This order can be attributed to the change in the zeta potential. AONs without drug loading had a potential of −40 mV, DOX-AONs had a potential of −36 mV, and PTX AONs had a potential of −21 mV. As the drug was encapsulated, the particle size increased. The lower the drug solubility in water, the lower the zeta potential value. LC and EE were determined using a UV-Vis spectrometer for DOX and HPLC for PTX. DOX showed an EE of 69.68% and an LC of 6.97%, and PTX showed an EE of 59.34% and a DL of 5.60%. DOX and PTX were successfully loaded into the AON. Furthermore, the drug release profiles from AONs were indicated in Figure S4. The burst release of drugs was minimized when they were encapsulated in AONs as compared to free drugs.

**Figure 1:**
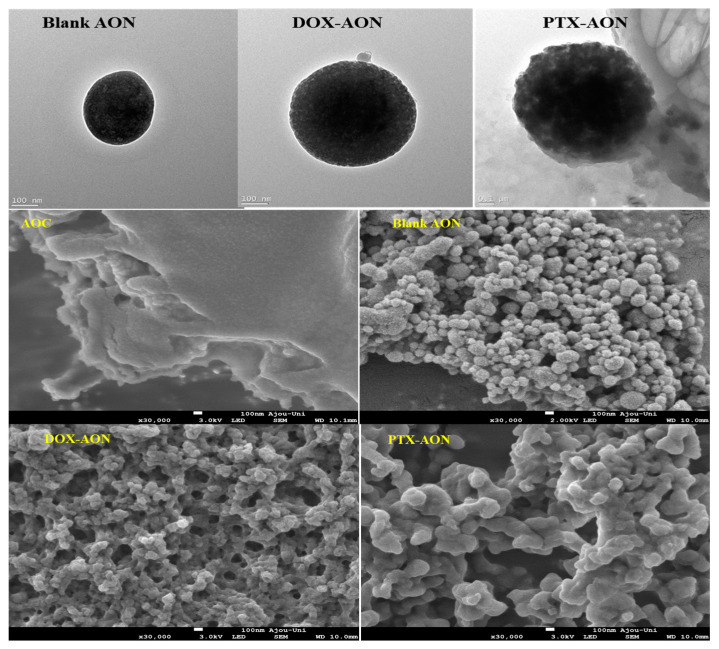
FE-TEM (**top**) and FE-SEM (**middle**, **bottom**) morphological images of blank AONs and drug-loaded AONs.

Figure 1 shows morphological images of (A) blank AONs, (B) DOX- AONs, and (C) PTX-AONs using FE-TEM (top) and FE-SEM (bottom). It was observed that the surfaces of the particles were not smooth and uneven when encapsulated. Because DOX is water-soluble (polar), the effect of surface charge is not significant, even when it interacts with AONs. However, poorly water-soluble PTX was dissolved in ethanol during the encapsulation in the AONs. During the encapsulation of PTX in AON, some denaturation of albumin may have occurred in ethanol [24]. However, PTX, a poorly soluble (non-polar) particle, was successfully encapsulated in albumin particles without denaturation but the surface charge of AONs was further lowered and becomes unstable [25]. Due to the low zeta potential of PTX-AONs, aggregation occurs between these particles, which are unstable on the surface.

### 2.2. Cellular Efficacy According to Shear Stress and Drug Type

**Figure 2:**
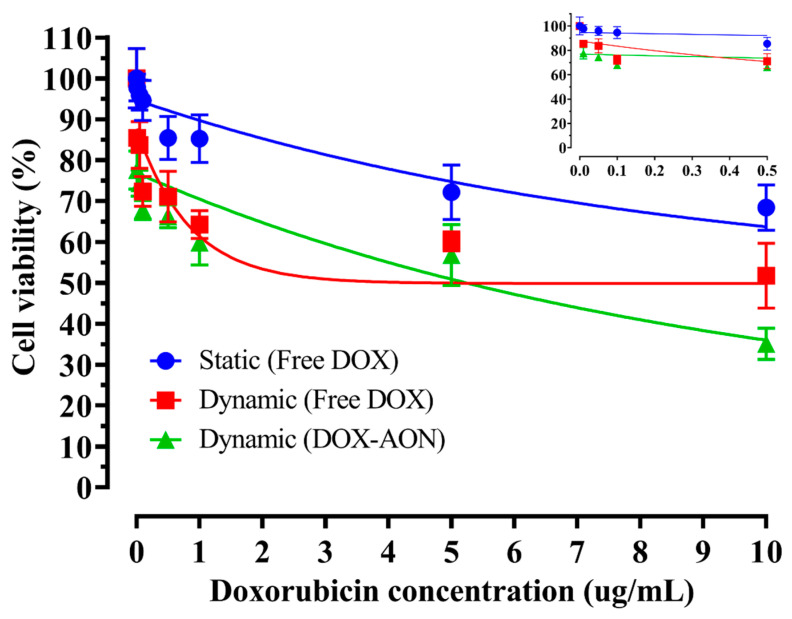
Cellular viability of free DOX and DOX-AONs as a function of drug concentration under static or dynamic conditions for 24 h (*n* = 4).

The cellular viability of free DOX and DOX-AONs under static and dynamic conditions as a function of DOX concentration is shown in Figure 2. After 24 h, the cellular viability in the dynamic condition was lower than that in the static condition. In addition, a higher decrease in cellular viability was observed when drug-loaded AON was compared to the free drug form under dynamic conditions.

**Figure 3:**
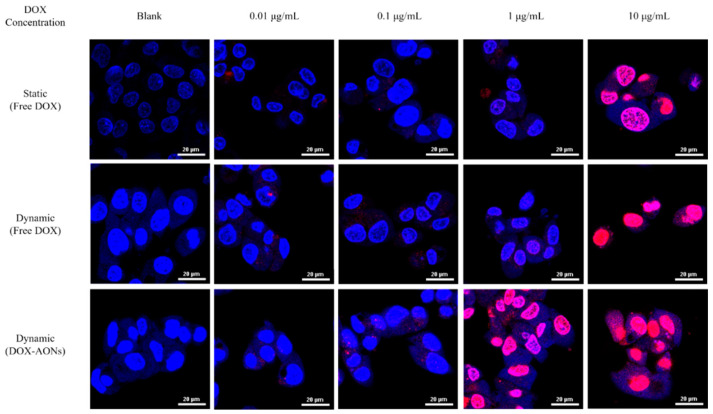
Observation of cellular images of internalized DOX and DOX-AONs under static and dynamic conditions using confocal laser scanning microscopy.

To observe the intracellular absorption patterns according to the shear stress and DOX-loaded nanostructure, the fluorescent images of internalized free DOX and DOX-AONs in cells under static and dynamic conditions using confocal scanning microcopy are given in Figure 3. When DOX was concentrated below 0.1 μg/mL, the degree of absorption into the cells of DOX under all culture conditions did not show a significant difference. However, at a concentration of DOX 1 μg/mL or more, the degree of fluorescence expression of DOX in cells was different according to culture conditions. When cells were exposed to free DOX, it was confirmed that DOX was moved more to the cell’s nucleus than under static conditions under dynamic conditions. In addition, under dynamic conditions of DOX 1 μg/mL or more, DOX-AONs were found to have a significantly greater degree of DOX fluorescence expression in the cell (cytoplasm, nucleus) than other culture conditions of the same concentration. Dynamic conditions are thought to have increased the cell absorption of DOX by increasing the expression of receptor protein genes in cells to promote the exchange of substances in cells. In addition, cells considered HSA and OA, which make up AON, as nutrients necessary for cell growth, and absorbed AON smoothly into the cell. These two phenomena were used to load DOX into AON, making it easier to absorb DOX into the cell than under other experimental conditions.

In particular, the increase in the absorption effect of DOX into the nucleus by AONs was observed. When DOX is encapsulated in AONs than its free drug formulation, it was absorbed into cells more effectively and binds to DNA in the nucleus and mitochondria, causing DNA damage and generating intracellular ROS to kill cancer cells due to oxidative stress [26]. The DOX encapsulated in AONs at the same concentration was more absorbed into the nucleus than the free drug formulation [27,28]. Based on these results, it can be inferred that drug-loaded AON can lower cellular viability and induce more ROS production than free drugs.

**Figure 4:**
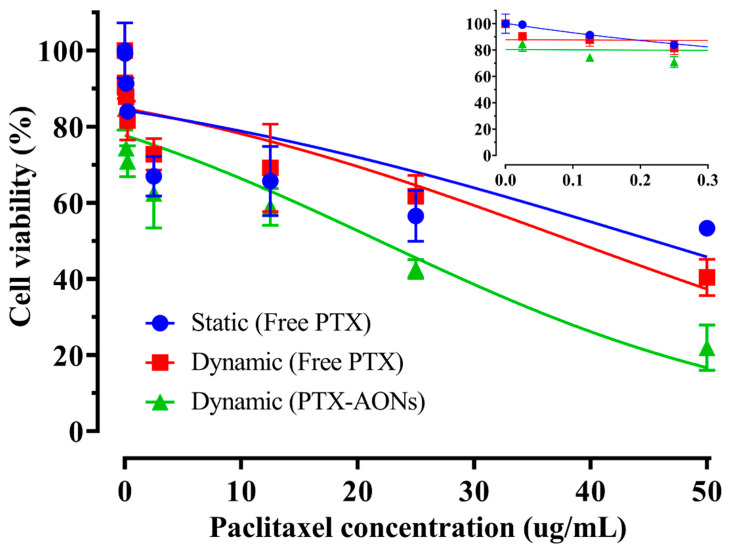
Cellular viability of free PTX and PTX-AONs as a function of drug concentration under static and dynamic conditions for 24 h (*n* = 4).

The cellular viability of free PTX and PTX-AONs as a function of drug concentration under static and dynamic conditions for 24 h is shown in Figure 4. After 24 h, it was found that the decrease in cellular viability was higher under dynamic conditions than that under static conditions. Under dynamic conditions, the decrease in cellular viability was much greater when AON was loaded in the free drug form. However, the extremely poorly water-soluble PTX it requires much higher drug concentration to have cell-killing effect as compared with highly water-soluble DOX.

The cellular efficacy of two anticancer model drugs with different drug solubility was determined by comparing the IC_50_ values according to the formulation and cell culture conditions. Table 2 shows the IC_50_ values (μg/mL) of free drugs and drug-loaded nanostructures (DOX-AONs, PTX-AONs) under static and hepatomimetic dynamic conditions (*n* = 4). In the case of DOX, the IC_50_ of free DOX was 13.4 μg/mL under static conditions and 11.798 μg/mL under dynamic conditions. The IC_50_ under dynamic conditions was 11.96% lower than that under static conditions. The IC_50_ value of DOX-AON under dynamic conditions was 5.613 μg/mL, which was 58.11% lower than that of free DOX under static conditions. Fluid flow in a dynamic environment and encapsulation of drugs in AONs reduced cellular viability. In contrast, the IC_50_ of free PTX was 45.44 μg/mL in the static condition, 38.43 ug/mL in the dynamic condition, and 21.86 μg/mL in the dynamic condition treated with PTX-loaded AON. The IC_50_ value of free PTX under dynamic conditions was reduced by 15.43% compared to that of free PTX under static conditions. The IC_50_ value of PTX-loaded AON under dynamic conditions was reduced by 51.89% compared to that of free PTX under static conditions. It was evident that cellular efficacy was well-correlated and highly dependent on the drug-loaded nanostructure, drug solubility, and physiological shear stress. The importance of drug solubility in the fattigated nanostructures was recognized in the cellular delivery and anticancer activity [16,29].

### 2.3. Real-Time ROS Sensing of Anticancer Drugs with Different Solubility

**Figure 5:**
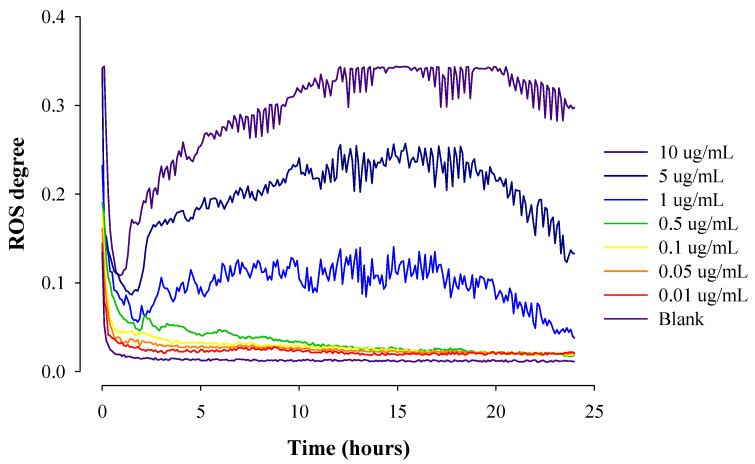
Real-time ROS occurrence from cells by varying DOX concentrations in HepG2 cells under dynamic conditions for 24 h (*n* = 3).

Figure 5 shows the real-time ROS occurrence from cells by varying DOX concentrations in HepG2 cells under dynamic conditions for 24 h. As the concentration of DOX increased, the degree of real-time ROS generation, i.e., the area under the ROS curves generated in cells treated significantly increased. For example, the calculated area (A.U) was 0.322, 0.554, 0.629, 0.696, 0.840, 2.340, 4.779, and 7.034 at the 0, 0.01, 0.05, 0.1, 0.5, 1, 5, and 10 μg/mL of DOX concentration, respectively.

**Figure 6:**
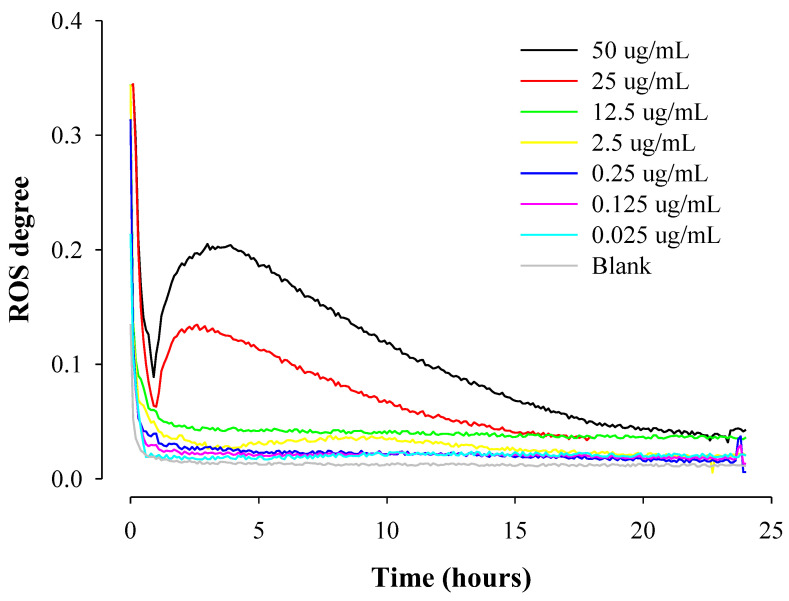
Real-time ROS occurrence from cells by varying PTX concentrations in HepG2 cells under dynamic conditions for 24 h (*n* = 3).

Figure 6 shows real-time ROS occurrence from cells by varying PTX concentrations in HepG2 cells under dynamic conditions for 24 h. As the concentration of PTX increased, the degree of real-time ROS generation, i.e., the area under the ROS curves generated in cells treated significantly increased. For example, the calculated area (A.U) was 0.322, 0.518, 0.528, 0.553, 0.725, 1.007, 1.685, and 2.586 at the 0, 0.025, 0.125, 0.25, 2.5, 12.5, 25, and 50 μg/mL of PTX concentration, respectively. PTX promotes ROS production by increasing the activity of nicotinamide adenine dinucleotide phosphate oxidase (NADPH oxidase; NOX) associated with the protoplasmic membrane of cells. The ROS produced in this way is known to induce abnormal actin polymerization in cells to inhibit tubulin binding, prevent cell division through microtubule stabilization, and induce cell apoptosis to exhibit anti-tumor effects [30,31,32,33]. ROS generated during this apoptosis process increased with increasing PTX concentration.

### 2.4. Correlation of Cellular Viability and Real-Time ROS Occurrence

**Figure 7:**
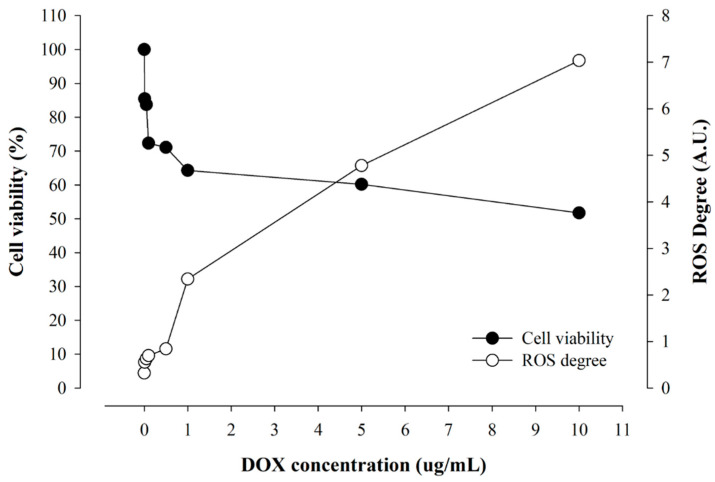
The coincidence of cellular viability and real-time ROS occurrence from cells by varying DOX concentrations.

**Figure 8:**
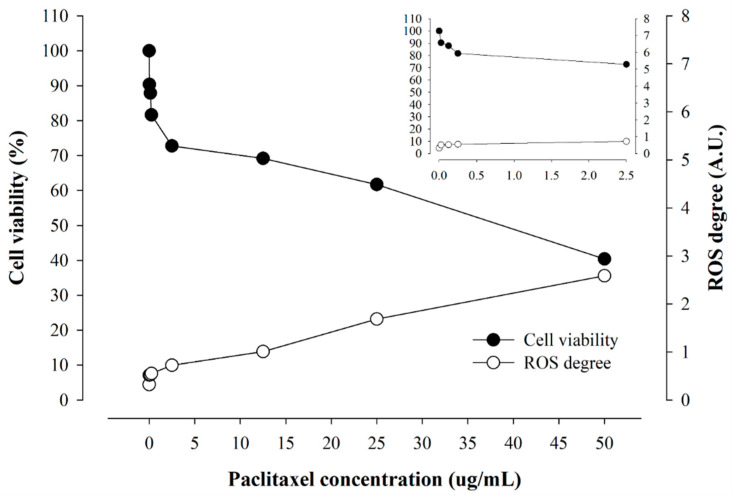
The coincidence of cellular viability and real-time ROS occurrence from cells by varying PTX concentrations.

The correlation of cellular viability and real-time ROS occurrence from cells as a function of drug concentration is shown in Figure 7 and Figure 8, respectively. As the DOX concentration increased, the cellular viability decreased, but the incidence of ROS increased accordingly. Notably, at the concentration of 1 μg/mL or less, the cellular viability decreased sharply as the concentration of DOX increased, but the increase in ROS was insignificant. The decrease in cellular viability was slowed with an increasing concentration above 1 μg/mL of DOX, and ROS increased rapidly. This resulted from the difference in the concentration of DOX inside and outside the cell affects the absorption rate of the drug. In addition, the degree of real-time ROS occurrence sharply increased as the degree of DOX binding to the DNA of the mitochondrial nucleus and the mitochondria described above increased.

Likewise, cellular viability decreased, but the occurrence of ROS increased as the PTX concentration increased. In PTX below 2.5 μg/mL, cellular viability decreased sharply as the concentration increased, but the increase in ROS was insignificant. In PTX 2.5 μg/mL or higher, cellular viability and ROS showed a gentle increase, showing a different tendency from DOX, which rapidly generated ROS at a certain concentration.

It was believed that the tendency of ROS occurrence by the two drugs resulting from cell killing effect of anticancer drugs was remarkably different because DOX and PTX have different mechanisms for generating ROS. It is known that DOX directly affects the mitochondria and nuclei of cells, leading to explosive ROS production inside the cell [26,34,35]. On the other hand, PTX inhibits microtubule synthesis and induces cells to collapse during cell proliferation, when ROS leaked from the mitochondria of the collapsed cells is believed to be measured by sensors [30,31,32,33]. In addition, the extremely poorly water-soluble PTX requires much higher drug concentration to generate ROS as compared with highly water-soluble DOX. However, it was evident that cellular efficacy and real-time ROS occurrence of two model drugs were well-correlated under the physiological shear stress.

## 3. Materials and Methods

### 3.1. Materials

Human serum albumin (HSA), oleic acid (OA), and DOX were purchased from Sigma-Aldrich (St. Louise, MO, USA). PTX was obtained from Daewoong Pharmaceutical Co., Ltd. (Seoul, Republic of Korea). 1-Ethyl-3-(3-dimethylamino-propyl) and N-hydroxysulfosuccinimide were purchased from Thermo Fisher Scientific Korea (Seoul, Republic of Korea). In contrast, biomimetic microfluidic system components were purchased from BioSpero Co., Ltd. (Jeju, Republic of Korea). Deionized water was processed using ultrapure water systems (Mirae ST Co., Ltd., Anyang, Republic of Korea). Other high-performance liquid chromatography (HPLC)-grade organic solvents were purchased from Samchundang Chemicals (Seoul, Republic of Korea).

### 3.2. Preparation of AONs, DOX-AONs, and PTX-AONs

#### 3.2.1. Synthesis of AOC

AOC for AON preparation was synthesized with some modifications according to the previous schematic protocols in Prof. Beom-Jin Lee’s Lab [36]. Briefly, 175 μL of OA were completely dissolved in 50 mL of dimethylformamide (DMF). Additionally, 103.5 mg 1-ethyl-3-(3-dimethylaminopropyl) carbodiimide (EDC) and 117.5 mg of sulfo-N-hydroxysuccinimide (sulfo-NHS) were added to the DMF in which OA was dissolved. This process was followed by magnetic stirring to ensure complete dissolution. After the reagents were completely dissolved, stirring was performed at a speed of 500 rpm at room temperature for 1 h to activate OA through the EDC-NHS coupling reaction. HSA (1 g) was weighed and stirred until it was completely dissolved in 100 mL of PBS (pH 7.4). Then, the activated OA solution was poured into the albumin solution and stirred at 37 °C for 24 h at a speed of 200 rpm. Then, 50 mL of acetone was added to the solution to precipitate AOC, and the solution was centrifuged at 10,000 rpm at 4 °C for 15 min. The supernatant was removed. The AOC was first washed twice with ethanol and washed once with deionized water to remove unreacted reagents (albumin and OA). AOC was dispersed in 40 mL of deionized water and poured into a dialysis membrane (Spectra/Por^®^ MWCO 12–14 kD Standard RC Dry Dialysis Kits, Spectrum Labs, San Francisco, CA, USA) for purification and dialyzed at room temperature for 24 h. The solution was then placed in a 50 mL tube and frozen in a deep freezer at −80 °C (I825UD48, Thermo Fisher Scientific, Waltham, MA USA) for six hours. This was followed by lyophilization using a freeze-dryer (FD8508, IlShinBioBase, Yangju-si, Gyeonggi-do, Republic of Korea) for at least two days.

#### 3.2.2. Conversion of AOCs to AONs Using a Desolvation Method

AOC (100 mg) was dispersed in 20 mL of 2-(N-morpholino) ethanesulfonic acid (MES) buffer to a concentration of 5 mg/mL. The AOC solution was stirred at 500 rpm, and 20 mL of methanol was added to the AOC solution at a rate of 1 mL/min. After mixing the solution, the resulting pellet was centrifuged at 10,000 rpm and 4 °C for 15 min. The pellet was placed in 30 mL of deionized water and placed in a dialysis membrane (12–14 kD) for 24 h. After dialysis was complete, the sample was placed in a 50 mL conical tube and frozen in a deep freezer for six hours. The frozen samples were subjected to lyophilization for two days to obtain AON powder.

#### 3.2.3. Preparation of Drug-Loaded AONs

Methods described in previous studies were utilized to encapsulate DOX in AONs [29]. AONs (10 mg) were dispersed in 10 mL of deionized water using an ultrasonic bath and vortex mixer. Then, 1 mg of DOX was weighed and placed in a solution in which the AONs were dispersed at 1 mg/mL. DOX was mixed using a vortex to encapsulate it in the AONs. Then, the mixed solution was incubated for three hours at 37 °C and 200 rpm using a stirring hot plate. Because DOX has photodegradable properties, it can decompose during incubation. The DOX-loaded nanoparticle solution was wrapped in aluminum foil to protect it from light during incubation.

A slightly modified version of a previous research method was used to encapsulate PTX in the AONs [37]. An amount of 10 mg of AONs was weighed and dispersed in 5 mL of deionized water. PTX (1 mg) was dispersed in absolute ethanol (5 mL) and added to the solution. The AONs were dispersed at a rate of 1 mL/min using a circulation pump. The mixed solution was incubated for 24 h at 37 °C and 100 rpm using a heat stirrer. The following procedure was performed to purify the drug-loaded AONs. The pellet was collected by centrifugation at 10,000 rpm at 4 °C for 15 min. The supernatant was discarded and washed three times with ethanol and deionized water to remove the remaining unencapsulated drug. DOX-AONs and PTX-AONs powders were obtained by vacuum drying for six hours.

### 3.3. Physicochemical Properties of NPs

#### 3.3.1. Fourier Transform-Infrared (FT-IR) Spectrometer

To confirm the conjugation synthesis between albumin and oleic acid, OA, HSA-OA physical mixture, and AOC powder were analyzed using an FT-IR spectrometer (Nicolet iS50, Thermo Scientific, Madison WI, USA). Each sample was checked in the wavelength range of 400–4000 cm^−1^ at a resolution of 2 cm^−1^.

#### 3.3.2. Particle Size and Zeta Potential Measurements

The particle size and zeta potential of blank AONs, DOX-AONs, and PTX-AONs were measured three times using a dynamic light scattering (DLS) instrument (ELSZ–2000 S, Otsuka Electronics, Osaka, Japan). Each sample was measured by dispersing it in deionized water at 5 mg/mL using an ultrasonicator and vortex mixer.

#### 3.3.3. Loading Content (LC) and Encapsulation Efficiency (EE)

Centrifugation was used to determine the amount of drug encapsulated in the AONs and each drug (DOX, PTX) remaining in the supernatant that was not encapsulated. DOX had a maximum absorbance of 480 nm and was analyzed using a UV/VIS spectrometer (DU730, Beckman Coulter, CA, USA). Before the analysis, we measured the degree of DOX absorption by concentration to confirm the calibration curve.

PTX had an absorbance maximum of 227 nm, and analysis was performed using HPLC. The HPLC system (Waters Alliance 2690, Waters Co., Milford, MA, USA) is a pump, UV-visible spectrophotometer detector (UV-996, Waters Co.), reversed-phase column (Gemini, C18 column, 150 × 4.6 mm, 5 μm, Phenomenex), and Integrator (Empower Pro software version 5.0). The PTX sample was centrifuged at 10,000 rpm for 10 min to remove residual AONs and impurities before HPLC analysis.

The mobile phase consisted of solution A and solution B (A:B = 30:70, *v*/*v*). Solution A was deionized water, and solution B consisted of acetonitrile. The flow rate was 1.0 mL/min, the injection volume was 50 μL, and the column temperature was adjusted to 30 °C. The analysis time for each sample was 20 min, and the PTX peak was confirmed at three minutes.

The LC and EE of the AONs were calculated using the following equations: The amount of drug loaded into the AONs was determined by dividing the amount of encapsulated drug by the weight of the initially added drug. LC was calculated by dividing the weight of the drug loaded into the AONs by the weight of the blank AONs to determine the ratio of the drug to the weight of the AONs.

(1) $$LC\left(\%\right)=\frac{Weight\;of\;the\;drug\;in\;AONs}{weight\;of\;AONs}\times 100$$

(2) $$EE\left(\%\right)=\frac{Amounts\;of\;the\;encapsulated\;drug\;in\;AONs}{Amounts\;of\;the\;initially\;added\;drug}\times 100$$

#### 3.3.4. Morphologies of NPs using FE-TEM and FE-SEM

The blank AONs (5 mg) or drug-loaded AONs (5 mg) were dispersed in 1 mL of deionized water by stirring at 200 rpm using a magnetic stirrer at room temperature. The sample was then coated with platinum once for 60 s in a vacuum by a sputtering machine (Cressington Q108 Auto Sputter Coater, Ted Pella, Redding, CA, USA). The morphological images were confirmed by FE-TEM (Tecnai G2 F30 S-Twin, Philips-FEI Corp., Best, The Netherlands).

For FE-SEM morphological images, twenty microliters of the sample were dropped onto a copper grid and stained with a 2% phosphotungstic (PTA) solution. The sample was dried at 37 °C for three hours to confirm the surface morphology using FE-SEM (JSM-7900F, JEOL, Tokyo, Japan).

### 3.4. Establishment of Biomimetic Microfluidic System with Real-Time ROS Sensor Chip

#### 3.4.1. Biomimetic Microfluidic System Calibration

**Figure 9:**
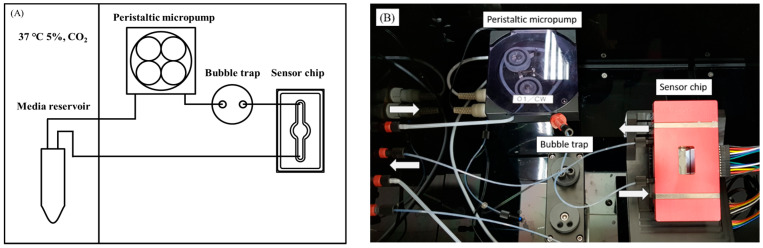
A schematic diagram of a biomimetic microfluidic system in which media circulate to simulate liver tissue and parts of the circulatory system. (**A**) Schematic diagram of the microfluidic components and the circulatory system and (**B**) photograph of a microfluidic system equipped with real-time generating ROS sensor chip (→ flow in, ← flow out).

**Figure 10:**
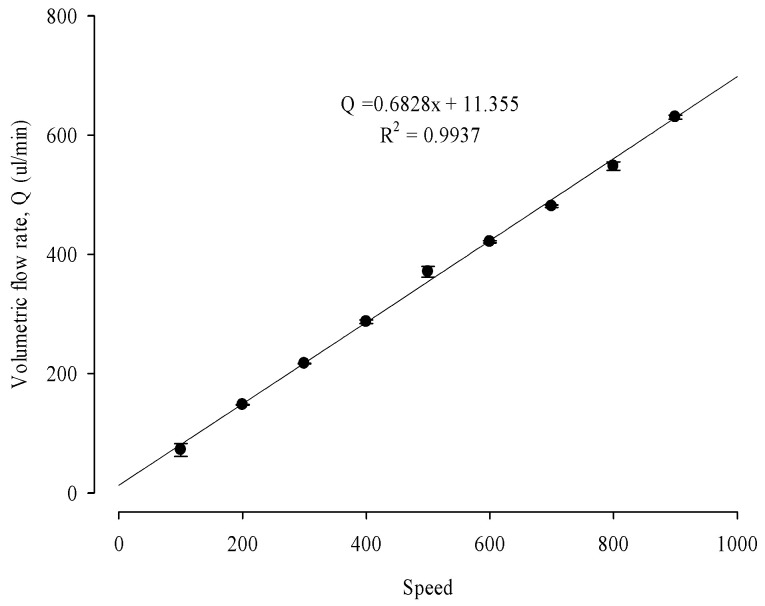
Standard calibration curve of a biomimetic microfluidic system between peristaltic pump speed and volumetric flow rate to determine the shear stress inside the sensor chip. The pump was operated for 1 min for each speed gauge, and the water weight delivered in the 1.5 microtubes was measured.

Shear stress was calculated by following a previous study to calibrate the microfluidic system for the shear stress level in the seeded cells in the chip. Figure 9 shows a schematic of the biomimetic microfluidic system. The gauge was set in units of 100 from 100–900, and deionized water was used for calibration. After setting the pump’s speed gauge, deionized water was flown for one minute each, and the weight of the deionized water was measured by placing it in a 1.5 mL Eppendorf tube. Each measurement was repeated three times. The weight of each empty EP tube was measured before the weight of the deionized water was measured to determine the weight difference. Figure 10 exhibited the standard calibration curve of the biomimetic microfluidic system between the peristaltic pump speed and the volumetric flow rate (Q). In the standard calibration curve of the microfluidic system between the pump speed and the volumetric flow rate (Q), the Q value increased linearly as the pump speed increased. Q can be extrapolated at a specific rate using the standard calibration equation (Q = 0.6828x + 11.355). Then, the shear stress (τ) was calculated at the fluid inlet of the chip using the Hagen–Poiseuille equation:

(3) $$\tau \left[dyne/{cm}^{2}\right]=\left(6\mu Q\right)÷\left(bh^{2}\right)$$

where μ is the viscosity (Pa·s) of the solution at 20 °C, 0.0010 for deionized water and 0.00096 for cell media; Q is the volume change in one minute, b, the width of the chip inlet is 0.29 cm, and h, the height of the inside of the chip is 0.05 cm, respectively.

Figure S1 shows visualized modeling to simulate the pressure (A) and flow velocity (B) and distribution of shear stress (C) applied inside the sensor chip using COMSOL Multiphysics^®^ software version 5.4. The pressure distribution values of the simulation presented coincided with the shear stress simulation modeling of the chip [7,38]. The standard of the ROS sensor chip was also confirmed and implemented in a COMSOL environment. The intensity of the shear stress is shown by the color variation where red denotes the highest shear stress while the darker blue color shows the lowest. The unit of the implemented model is Pa, which corresponds to 1 Pa = 10 dyne/cm^2^. Therefore, the flow shear stress corresponding to 5 dyne/cm^2^ in physiological liver cells was applied since the implemented model chip has a pressure of 0.5 Pa at the center. When the circulation pump speed (x) was set to 60, the volumetric flow rate (Q) was 0.05323 mL/min. The shear stress (τ) at the center of the chip as dynamic conditions was optimally set 5 dyne/cm^2^ because the fluidic shear stress in the liver ranged from approximately 1–10 dyne/cm^2^ [39,40].

#### 3.4.2. Cell Culture in a Real-Time ROS Sensor Chip

The human hepatocellular carcinoma cell line HepG2 was obtained from the Korean Cell Line Bank (KCLB No. 88065, Seoul, Republic of Korea). HepG2 is a suitable cell line for evaluation, as it is a liver cancer cell line [39,40]. The cell was used to form a chip corresponding to the liver. The cells were cultured in DMEM supplemented with 10% fetal bovine serum and 1% penicillin/streptomycin. The cells were seeded on the chip for sensor chip-based microfluidic experiments. The collagen coating was performed before cell seeding. Next, 246 μL of pH 7.4 PBS and 4 μL of collagen stock (2 mg/mL 0.02 N acetic acid and collagen 1 high concentration rat tail, Corning, NY, USA) were added to the seeding kit. This was followed by UV irradiation for 10 min. After that, the coating process was performed in an incubator at 37 °C and 5% CO_2_ for 30 min. The collagen solution was removed, and the seeding kit was washed twice with 200 μL of PBS to remove any remaining collagen. A cell solution (200 μL) adjusted to 3 × 10^5^ cells/mL was added to the seeding kit and incubated in an incubator for two hours to allow cells to adhere to the sensor chip. The cultured HepG2 cells did not result in any alterations in their cellular properties under dynamic conditions.

The whole microfluidic system and real-time ROS sensor chip used a prototype manufactured by Jeju National University (Jeju, Republic of Korea). Detailed information on the sources of materials, preparation method, and physical properties of the microfluidic system and ROS chip were described more clearly in Supplementary S1.1 on the Fabrication and Physical Properties of Microfluidic Chip Having Real-time ROS Chip Sensor. Briefly, a sensor chip is made of glass, and a frame consisting of polydimethylsiloxane (PDMS) is attached to the inside of the chip to allow fluid to flow. In preliminary study, the two anticancer model drugs had no adsorption to PDMS devices during microfluidic experiments.

#### 3.4.3. Cellular Viability Assay

To check cell efficacy and toxicity in diseased cells, a chip in which cells were seeded at 3 × 10^5^ cells/mL was mounted in a microfluidic system. Then DOX (0.01, 0.05, 0.1, 0.5, 1, 5, or 10 μg/mL) or PTX (0.025, 0.125, 0.25, 2.5, 12.5, 25, or 50 μg/mL) was analyzed in free drug and loaded AONs at different concentrations. The concentration range of the drug was set by referring to in vitro, in vivo, and clinical trials for each drug [41,42,43,44]. When PTX was added to the medium, it was dissolved in DMSO, and the volume was adjusted so that DMSO did not exceed 1% of the total medium volume (e.g., if the final medium volume was 5 mL, the DMSO in which PTX was dissolved was adjusted to 50 μL). Cells were exposed to static and dynamic conditions (5 dyne/cm^2^) for 24 h in a serum-free medium containing the drug. A microfluidic system was used to circulate the drug-containing medium.

Cell viability was evaluated using the MTT assay. After unmounting the chip from the microfluidic system, the cells were washed with PBS to remove any remaining drug. The cells were then treated with 140 μL of fresh serum-free medium containing 10% MTT solution. The cells were incubated for one hour at 37 °C in 5% CO_2_. After removing the medium, 140 μL of DMSO was added to perform pipetting until the formazan crystals were completely dissolved. The completely dissolved formazan solution was transferred to a 96-well plate, and the absorbance was measured at 570 nm using a microplate reader (Synergy H1, BioTek, Santa Clara, CA, USA). The samples for each concentration were repeated three times, and the apoptosis results were normalized based on the cells exposed to the serum-free medium not treated with the drug. IC_50_ values were calculated using GraphPad Prism software (Version. 9, San Diego, CA, USA).

#### 3.4.4. Real-Time Sensing of the Generated ROS

**Figure 11:**
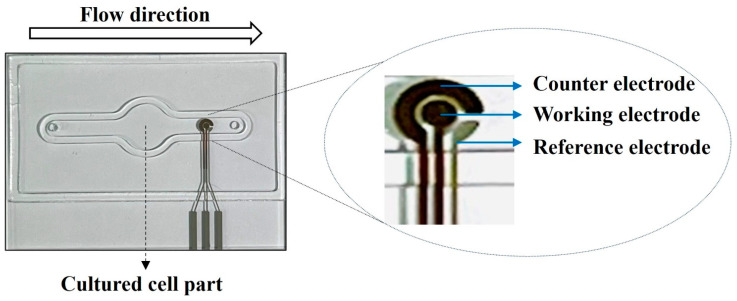
Photographic images of the electrode for measuring real-time ROS from cells exposed to drugs. A three-electrode system was adopted for electrodes based on the cyclic voltammetry method. The generated ROS oxidized working electrodes to induce electrical stimulation to measure the degree of ROS occurrence quantitatively.

A sensor chip for detecting ROS generated in cells was printed on a glass chip using a solution-based inkjet printing technique by a multi-head 3D printer [45]. Electrodes capable of detecting ROS were printed using an inkjet injection method on the chip using three electrode systems (working electrode, counter electrode, reference electrode) [46]. The working electrode and counter electrode were printed in gold ink, and the reference electrode was printed in silver ink. The ROS generated in cells was quantitatively measured through a faradaic redox reaction in electrodes using the cyclic voltammetry (CV) method. The ROS oxidized the working electrode of the sensor, to which the CV method was applied to induce electrical stimulation. Based on the degree of electrical stimulation caused by the ROS, the generated ROS occurrence could be quantitatively measured. Figure 11 shows the photographic images of the electrode for measuring real-time ROS from cells exposed to drugs.

The sensor chip in which the cells were seeded was mounted to the microfluidic system, and a humidified 37 °C, 5% CO_2_ environment was created in the drug-containing medium (except for the pump and bubble trap). The culture medium containing the drug flowed into the chip so that the cell monolayer was subjected to shear stress using a peristaltic pump. The pump speed was adjusted, so the shear stress on the cells in a dynamic condition was 5 dyne/cm^2^. For comparison, the cells were exposed to serum-free and drug-free media in a static environment without microfluidic flow and serum-free medium-containing drugs. The ROS generated in the cells was measured at six-minute intervals for 24 h, and the exposed sensor chip of the light source was covered with aluminum foil to prevent it from being exposed to light. To quantify the ROS caused by exposing cells to drugs, the area at the bottom of the ROS graph by drug concentration was calculated. During measurement by the sensor, the ROS value changes over time. The difference in ROS values generated by the concentration of the drug may vary depending on the specific point in time that is arbitrarily set, so the correlation between cellular viability and ROS may be interpreted each time differently. Therefore, comparing the total amount of ROS that occurred during the time cells were exposed to drugs was considered more appropriate to interpret the correlation between the drug-induced cell survival rate and the degree of ROS occurrence.

### 3.5. Cellular Images Using Confocal Laser Scanning Microscopy

The experimental procedure and pre-treatment of the sample were performed with some modifications, such as those of a previous study [37]. Cells were exposed to static and dynamic environments in a DOX-AONs-treated serum-free culture medium at 37 °C and 5% CO_2_ for one hour. The cells were washed with 150 μL of PBS for five minutes to remove the remaining culture medium and DOX-AONs. 0.4 Trypan blue solution (0.4%) was treated for 5 min and washed twice with PBS for 5 min each to quench the fluorescence outside the cell. To fix the cells, they were treated with 4% paraformaldehyde solution for 10 min and washed twice with PBS for five minutes each. Hoechst #33342 solution was added for 15 min to stain the cell nuclei and washed with PBS for five minutes. All washing processes were performed at 37 °C and 5% CO_2_. Then, PBS was filled, and the cells were observed via fluorescence microscopy (Eclipse A1Rsi and Eclipse Ti-E, Nikon, Melville, NY, USA).

## 4. Conclusions

In this study, we assessed the cellular efficacy of albumin-oleic acid nanoparticles encapsulating two model anticancer drugs, hydrophilic DOX and hydrophobic PTX. Cellular viability and real-time ROS occurrence of the free drug or drug-loaded AON using a biomimetic microfluidic chip system were investigated. The IC_50_ values of two model drugs was highly affected by dynamic shear stress and by encapsulating drug in AONs, giving increased anticancer efficacy and ROS occurrence. Furthermore, DOX with high water solubility showed better cell-killing effect and higher ROS occurrence at the same concentration. There was also a good coincidence of cellular viability and real-time ROS occurrence. It was also observed that cellular viability and real-time ROS occurrence were highly dependent on the drug solubility and shear stress. The current sensor chip-based biomimetic systems could be a valuable tool for monitoring real-time drug efficacy and predicting drug toxicity of diverse model drugs using human-oriented tissues and organs with minimizing animal sacrifice. Furthermore, multi-connected organ-on-chips including current hepatomimetic chip can be used for determining pharmacokinetics and drug bioavailability to replace costly and time-consuming animal experiments.

## Figures and Tables

**Table 1:** Physicochemical properties of self-assembled AONs with or without drug loadings (*n* = 3).

	Particle Size (nm)	Polydispersity Index	Zeta Potential (mV)	Loading Content (%)	Encapsulation Efficiency (%)
AONs	181.20 ± 29.20	0.300 ± 0.005	−40.57 ± 0.22	-	-
DOX-AONs	313.23 ± 3.97	0.180 ± 0.024	−36.36 ± 0.10	6.97 ± 0.33	69.68 ± 3.26
PTX-AONs	438.90 ± 27.97	0.170 ± 0.081	−21.04 ± 0.16	5.60 ± 0.14	59.34 ± 1.56

**Table 2:** Half-maximal inhibitory concentration (IC_50_) values (μg/mL) of free drugs and drug-loaded nanostructures (DOX-AONs, PTX-AONs) under static and hepatomimetic dynamic conditions (*n* = 4).

Formulation	Shear Stress	IC_50_ Values (μg/mL)
Free DOX	Static	13.4 ± 1.345
Free DOX	Dynamic	11.798 ± 1.721
DOX-AONs	Dynamic	5.613 ± 1.601
Free PTX	Static	45.44 ± 5.824
Free PTX	Dynamic	38.43 ± 3.123
PTX-AONs	Dynamic	21.86 ± 2.340

## Data Availability

Data are contained within the article. Data will be made available on request.

## Supplementary Materials

The following supporting information can be downloaded at: https://www.mdpi.com/article/10.3390/ph16091330/s1, S1.1: Fabrication and Physical Properties of Microfluidic System Having Real-time ROS Chip Sensor; Figure S1: Visualized modeling to simulate the pressure (A) and flow velocity (B) and distribution of shear stress (C) applied inside the sensor chip; Figure S2: Fourier-transform infrared (FT-IR) spectra of oleic acid (OA), human serum albumin (HSA), physical mixture (HSA and OA), and HSA-OA conjugate (AOC); Figure S3: Particle distribution of (A) albumin-oleic acid nanoparticles (AONs) (181.20 ± 29.20 nm), (B) doxorubicin (DOX)-AONs (313.23 ± 3.97 nm), and (C) paclitaxel (PTX)-AONs (438.90 ± 27.97 nm); Figure S4: Release profiles of free drug and drug-loaded albumin-oleic acid nanoparticles (AONs) in PBS solution (pH 7.4).
