# Supplementary material for: Cellular Efficacy of Fattigated Nanoparticles and Real-Time ROS Occurrence Using Microfluidic Hepatocarcinoma Chip System: Effect of Anticancer Drug Solubility and Shear Stress
Source: Pharmaceuticals (Basel). 2023 Sep 20;16(9):1330. doi: 10.3390/ph16091330 (PMC10536289; doi:10.3390/ph16091330)
Supplement: Supplementary file 1 [file pharmaceuticals-16-01330-s001.zip › pharmaceuticals-2591428-supplementary.pdf]

S1.1. Fabrication and Physical Properties of Microfluidic System Having Real-time ROS Chip Sensor

Microfluidic chips are constructed using soda lime glass sheets with pre-printed ROS electrodes. The top and bottom chips had dimensions of 41 mm width, 1.1 mm thickness, and 56 mm length. The design was to create a channel with a volume of 64.271 mm^3 and a surface area of 457.15 mm^2. The microfluidic channels on the inside surface of the top glass were printed with Nusil medical grade silicone (MED-6033) elastomer using an inkjet printer. Preparation of the silicone elastomer was performed according to the manufacturer's protocol, and the material was degassed before loading into the 3D printer nozzle.

The microfluidic channels were 800 μm wide and 300 m tall. To establish fluid circulation, microfluidic connectors were fixed on the top glass. To assemble the glasses into the form of a hepatomimetic microfluidic system having real-time ROS chip sensor, a magnetic chip holder was created. The microfluidic chip was sterilized for 15 minutes with a 70% ethanol solution after being rinsed three times with 1X PBS. The chip is made up of transparent glass, and the experiment is performed inside an opaque microphysiological system that follows the principle of a cell culture incubator, maintaining 37 ^{o}C temperature, 5 % CO_2, 21% Oxygen in dark conditions.

The chip is only exposed to light only while the process of media changes and microscopic observation. which is in an insignificant amount of time. There is no air permeability within the microfluidic chip, the only oxygen content in the media is present in the form of dissolved oxygen. The air-tight chip protects from contamination and exchange of air into the media from the outside environment. In comparison to conventional 2D in-vitro cell culture experiments, the chip system is more relative to real human tissue physiology as it follows the dynamic fluid flow hence leading to better cell viability and molecular profiles of biomarkers.

The shear stress modeling inside the chip is provided in Figure S1. The conditions and dimensions for the shear stress simulation are kept the same, except it is done in 2D. The shear stress distribution observed in the chip is validated as done in [7,38]. The resulting values range from 1 to 8×10^{-3} with a standard unit of Pascal (Pa). The intensity of the shear stress is shown by the color variation where red denotes the highest shear stress while the darker blue color shows the lowest. The pressure is measured in Pa. The color variation here also shows the density of pressure, as done in [38]. The pressure distribution values of the simulation presented in Figure 10 coincided with Figure S1 presented in [38].

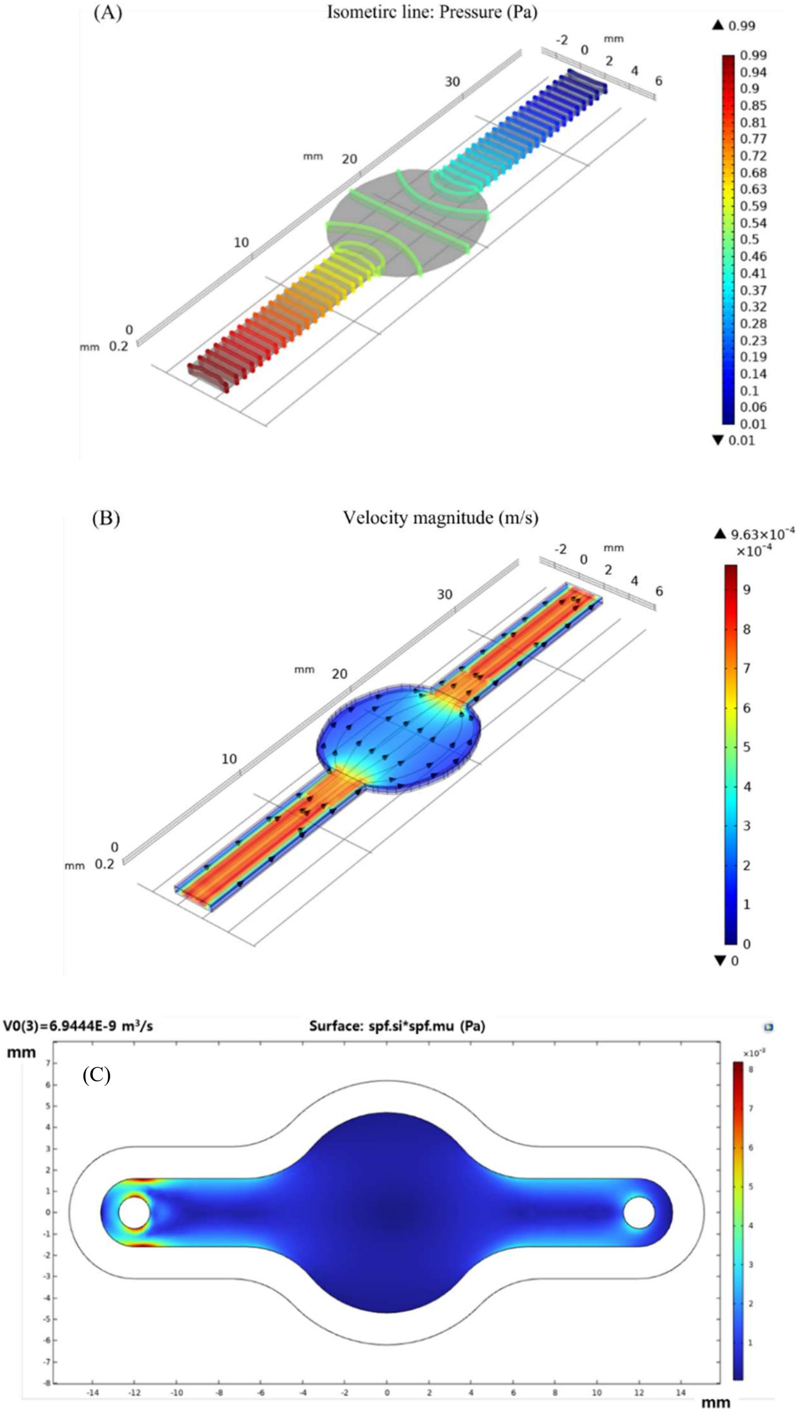

Figure S1. Visualized modeling to simulate the pressure (A) and flow velocity (B) and distribution of shear stress (C) applied inside the sensor chip

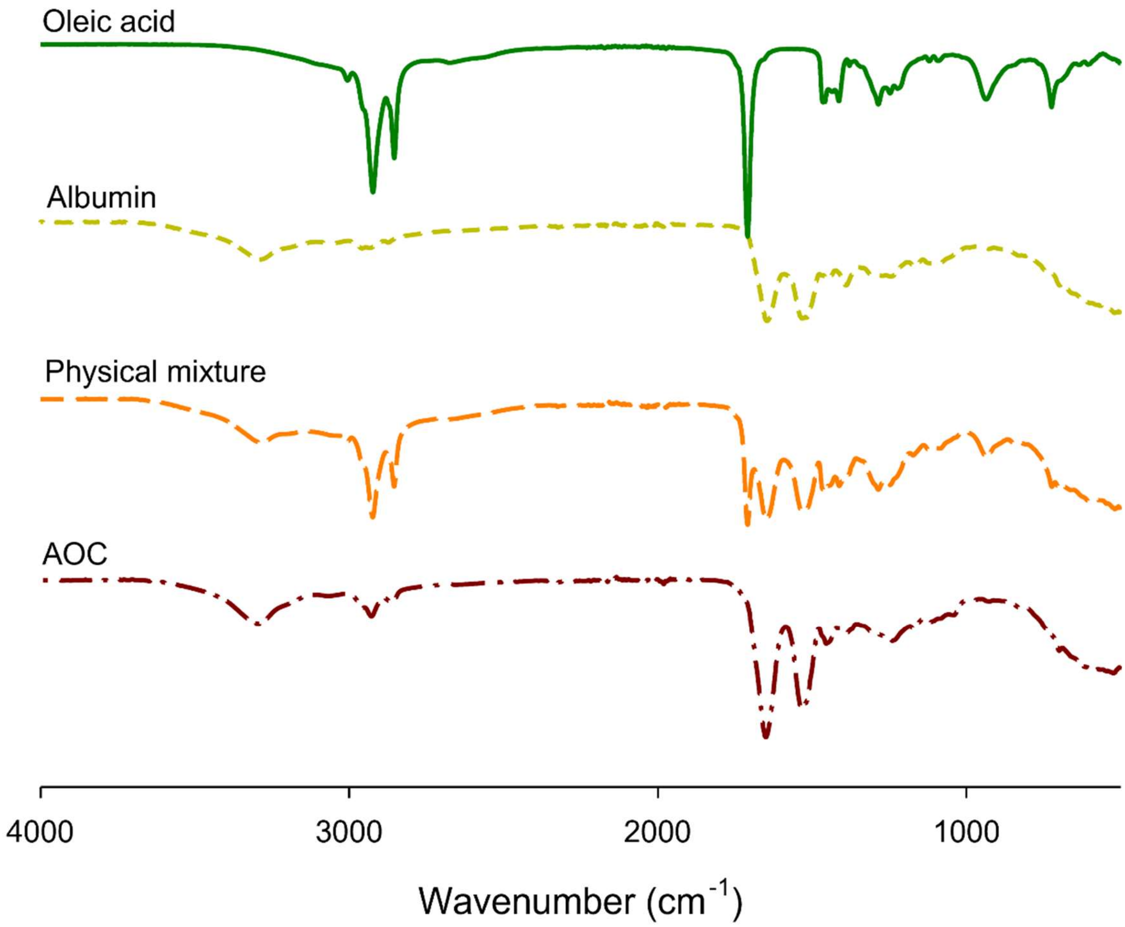

Figure S2. Fourier-transform infrared (FT-IR) spectra of oleic acid (OA), human serum albumin (HSA), physical mixture (HSA and OA), and HSA-OA conjugate (AOC).

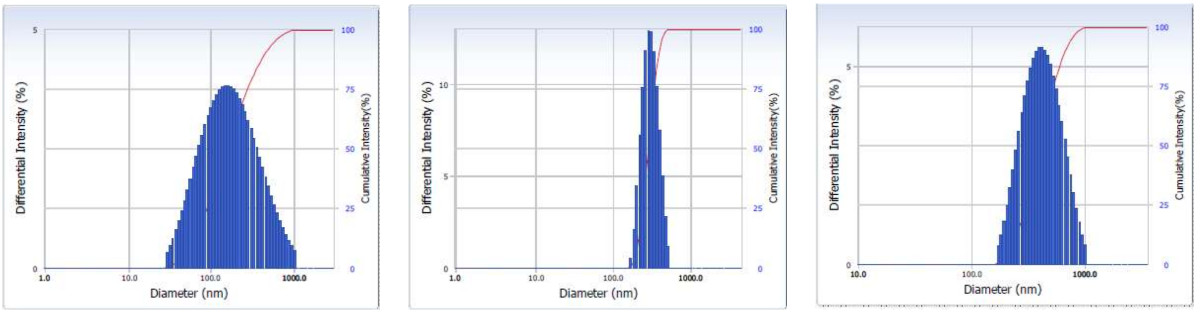

(A) (B) (C)

Figure S3. Particle distribution of (**A**) albumin-oleic acid nanoparticles (AONs) (181.20 ± 29.20 nm), (**B**) doxorubicin (DOX)-AONs (313.23 ± 3.97 nm), and (**C**) paclitaxel (PTX)- AONs (438.90 ± 27.97 nm).

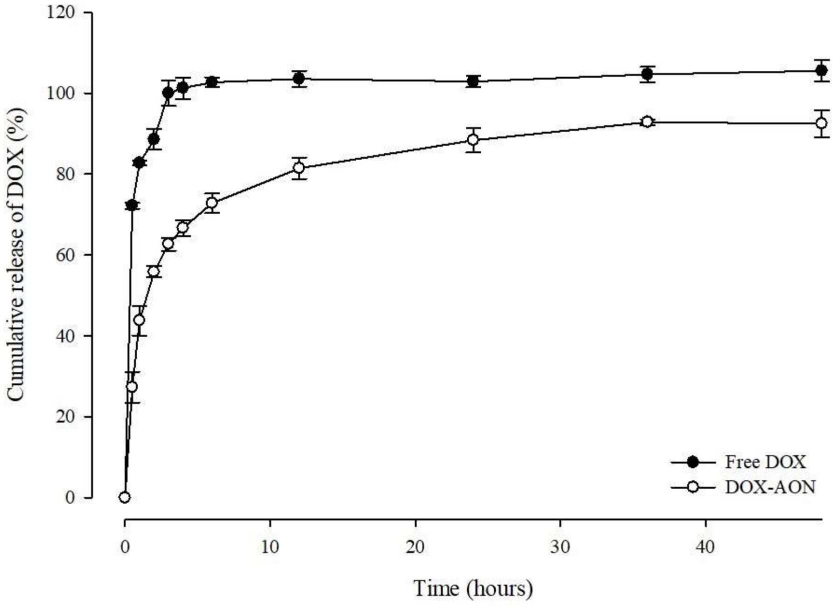

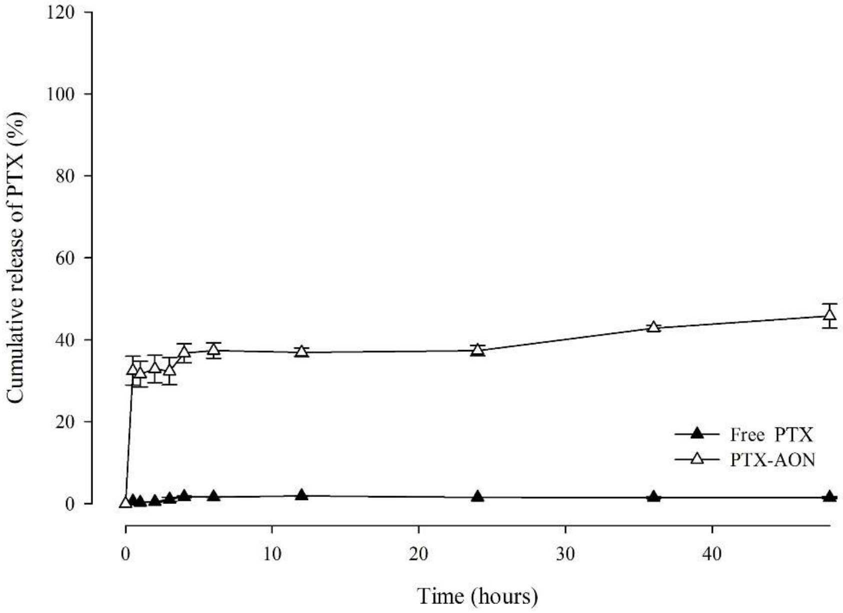

Figure S4. Release profiles of free drug and drug-loaded albumin-oleic acid nanoparticles (AONs) in PBS solution (pH 7.4).

References

1. Pisapia, F.; Balachandran, W.; Rasekh, M. Organ-on-a-Chip: Design and simulation of various microfluidic channel geometries for the influence of fluid dynamic parameters. *Appl. Sci.* **2022**, *12*, 3829.
2. Filipovic, N.; Nikolic, M.; Sustersic, T. Simulation of organ-on-a-chip systems. In *Biomaterials for Organ and Tissue Regeneration*; Elsevier: Amsterdam, The Netherlands, 2020; pp. 753–790.
